# Chronic Central Administration of Ghrelin Increases Bone Mass through a Mechanism Independent of Appetite Regulation

**DOI:** 10.1371/journal.pone.0065505

**Published:** 2013-07-02

**Authors:** Hyung Jin Choi, Kyoung Ho Ki, Jae-Yeon Yang, Bo Young Jang, Jung Ah Song, Wook-Young Baek, Jung Hee Kim, Jee Hyun An, Sang Wan Kim, Seong Yeon Kim, Jung-Eun Kim, Chan Soo Shin

**Affiliations:** 1 Department of Internal Medicine, Seoul National University College of Medicine, Seoul, Korea; 2 Department of Molecular Medicine, Cell and Matrix Research Institute, Kyungpook National University School of Medicine, Daegu, Korea; Clermont Université, France

## Abstract

Leptin plays a critical role in the central regulation of bone mass. Ghrelin counteracts leptin. In this study, we investigated the effect of chronic intracerebroventricular administration of ghrelin on bone mass in Sprague-Dawley rats (1.5 μg/day for 21 days). Rats were divided into control, ghrelin *ad libitum*-fed (ghrelin *ad lib*-fed), and ghrelin pair-fed groups. Ghrelin intracerebroventricular infusion significantly increased body weight in ghrelin *ad lib*-fed rats but not in ghrelin pair-fed rats, as compared with control rats. Chronic intracerebroventricular ghrelin infusion significantly increased bone mass in the ghrelin pair-fed group compared with control as indicated by increased bone volume percentage, trabecular thickness, trabecular number and volumetric bone mineral density in tibia trabecular bone. There was no significant difference in trabecular bone mass between the control group and the ghrelin ad-lib fed group. Chronic intracerebroventricular ghrelin infusion significantly increased the mineral apposition rate in the ghrelin pair-fed group as compared with control. In conclusion, chronic central administration of ghrelin increases bone mass through a mechanism that is independent of body weight, suggesting that ghrelin may have a bone anabolic effect through the central nervous system.

## Introduction

The brain is regarded as the master regulator of homeostasis and metabolism. It has been suggested that bone and energy metabolism require tightly coordinated regulation so that longitudinal growth, or bone remodeling, are in accordance with energy supply and demand [1]. Numerous studies have investigated the mechanisms involved in the central regulation of bone metabolism and possible connections between bone metabolism and energy metabolism [2]–[7]. However, the data are contradictory regarding bone metabolism regulation. In addition, the factors that co-regulate bone metabolism and energy metabolism are not yet clear.

Leptin, a major adipokine that regulates appetite, has been widely investigated as the major factor co-regulating bone metabolism and energy metabolism [6]. Ducy et al. demonstrated that intracerebroventricular (ICV) injection of leptin induced bone loss in the spine, suggesting a role for leptin in bone metabolism regulation through a central mechanism [6]. Data from subsequent reports supported the bone catabolic effect of leptin and have shown that leptin inhibits bone formation via the sympathetic nervous system [4], [5]. However, several recent studies have reported contradicting data [2], [3], [7]. Leptin administration via gene therapy into the hypothalamus did not have a significant effect on bone metabolism [3]. Leptin receptor deficient mice exhibit decreased bone mass, demonstrating leptin's anabolic effect on bone [7]. Furthermore, ICV injection of leptin increases bone mineral density (BMD) and mineral apposition rates [4], a finding which is the exact opposite of the initial studies reporting a catabolic effect of central leptin [6]. Given this conflicting data, further studies are required to clarify the mechanisms underlying the central regulation of bone metabolism.

Ghrelin is a stomach hormone that acts centrally to increase appetite [8]. Several studies have investigated the effect of chronic ICV ghrelin on energy metabolism. Chronic ICV ghrelin infusion increased food intake and weight gain [8], reversed the effect of leptin on food intake [9], increased the glucose utilization rate of adipose tissue [10], and increased lipogenic enzymatic activity in adipose tissue and liver [11]. A recent study investigated the central effects of ghrelin and leptin on body and bone marrow adiposity, as well as adipose tissue and bone marrow gene expression, and reported central ghrelin had no effect on bone marrow adiposity [12]. However, to date, no study has investigated the effect of chronic ICV ghrelin on bone metabolism.

Ghrelin and leptin have opposite effects on energy metabolism, but also on the sympathetic nervous system and other pathways [13]–[15]. Central leptin stimulates sympathetic outflow [13] whereas central ghrelin suppresses sympathetic activity [14]. Since the sympathetic nervous system has been suggested as the major pathway governing the effect of central leptin on bone metabolism [6], it is possible that sympathetic suppression through central ghrelin can affect bone metabolism. Therefore, the present study was designed to investigate the chronic effects of centrally administered ghrelin on bone metabolism.

## Materials and Methods

### Animals and peptide

Male Sprague-Dawley rats (6 weeks old) weighing 180–230 g were used. Body weight and food intake were monitored daily. All animal experiments were performed with approval from the Institutional Animal Care and Use Committee (IACUC) of the Clinical Research Institute at Seoul National University Hospital (an AAALAC accredited facility). National Research Council (NRC) guidelines for the care and use of laboratory animals were also observed (1996 revision). The standard rodent chow (Purina Rodent Chow; Biopia, Korea) was used. Ghrelin (rat) was purchased from Bachem Inc. (Bubendorf, Switzerland).The ghrelin peptide was prepared with concentration of of 0.25 µg/µl which corresponds to 1.5 µg/day (6 µg/day; 7.14 µg/Kg body weight/day).

### Surgery

Rats were anesthetized by intraperitoneal injection of 50 mg/kg zoletil and 10 mg/kg xylazine and surgically implanted with a 22-gauge stainless-steel cannula (Plastics One Inc. Roanoke, VA, USA) into the third cerebral ventricle. Osmotic mini-pumps (Model 2004, 0.25 μL/h; Alzet Corp., Cupertino, CA, USA) filled with saline or rat ghrelin peptide were implanted under the dorsal chest skin. The mini-pumps were connected to the ICV cannula via a catheter. Alzet Brain infusion kit 2 with infusion cannula of ID  = 0.18 mm and OD  = 0.36 mm was used. The cannula was stereotactically placed 0.72 mm posterior to the bregma on the midline and implanted 7 mm below the outer surface of the skull surgery via a stereotaxic apparatus. For the verification for correct cannula placement, cresyl violet staining and brain dissection was performed.

### Study design

Fifteen rats were divided into 3 groups: the control group (n = 5) received ICV infusions of saline for 21 days; the ghrelin *ad libitum*-fed (ghrelin *ad lib*-fed) group (n = 4) received ICV infusions of ghrelin (1.5 μg/day) for 21 days; the ghrelin pair-fed group (n = 6) received ICV infusions of ghrelin (1.5 μg/day) for 21 days and were allowed to eat only as much chow as consumed by the control group on the previous day.

### Radiological analyses and bone histomorphometry

Nondestructive, three-dimensional evaluation of bone mass and architecture were performed using a microCT scanner (Skyscan 1076 for tibia and lumbar spine and Skyscan 1172 for femur; Skyscan, Aartselaar, Belgium). Lumbar spine and femur were dissected from soft tissue, fixed in 70% ethanol, and analyzed. Image acquisition of tibia and lumbar spine L3 was performed with a source voltage of 100 kV, current of 100 μA, a 0.5-mm aluminum filter, and an isotropic voxel size of 8.8 μm. Image acquisition of femur was performed with a source voltage of 70 kV, current of 141 μA, a 0.5-mm aluminum filter, and an isotropic voxel size of 11.55 μm. For the tibia metaphysis trabecular bone, 251-slice-thick volume of interest starting 150 slices distal to the proximal growth plate were analyzed. For the tibia diaphysis cortex, the mid-diaphysis cortical bone volume of interest was analyzed. For the femoral metaphysis, 101-slice-thick volumes of interest starting 150 slices proximal to the distal growth plate were analyzed. For the lumbar spine, trabecular bone volumes of interest of whole trans-axial spine body images were analyzed. For trabecular volumetric bone mineral density (BMD) analyses using microCT, phantoms with predefined densities and CTA software were used for BMD measurements, as described in the manufacturer's manual (Skyscan, Aartselaar, Belgium). The trabecular and cortical bone volumes of interest were outlined by interpolation of operator-drawn regions exclusively representing the trabecular and cortical bone, respectively.

BMD (g/cm^2^) of the *ex vivo* tibia, femur, and lumbar spine (L2 and L3) were measured using the dual energy X-ray absorptiometry (DXA) instrument PIXIMUS (GE Lunar, Madison, WI, USA). A phantom was scanned daily for quality control.

For histomorphometric analyses of dynamic parameters, rats were injected with calcein (20 mg/kg body weight) 2 and 6 days prior to sacrifice. Dynamic histomorphometry analyses were conducted on the lumbar spine L5 using the Bioquant program (Bio-Quant. Inc., San Diego, CA, USA) [16].

### Serum marker analyses

The serum concentrations of procollagen type 1 amino-terminal propeptide (P1NP), cross-linked C-telopeptide (CTX), and tartrate-resistant acid phosphatase 5 b (TRAP-5b) were determined by ELISA, following the manufacturer's protocol (Immunodiagnostic Systems Inc, Scottsdale, AZ, USA). The serum concentrations of ghrelin and leptin were determined by ELISA, per the manufacturers' protocols (Linco Research, St Charles, MO, USA and Millipore, Billerica, MA, USA, respectively). Serum insulin-like growth factor 1 (IGF-1) was determined by immunoenzymometric assay (IEMA) following the manufacturer's protocol (GroPep-IDS, Fountain Hills, CA, USA).

### Statistical Analyses

Data are presented as the mean ± SEM. Statistical analyses were performed using analysis of variance (ANOVA) with least significant difference (LSD) as a *post hoc* comparison. Significance was defined as P<0.05. Statistical analyses were performed with SPSS for Windows (version 17.0, SPSS Inc., Chicago, IL, USA).

## Results

### Chronic ICV ghrelin infusion increases body weight and food intake

**Figure 1:**
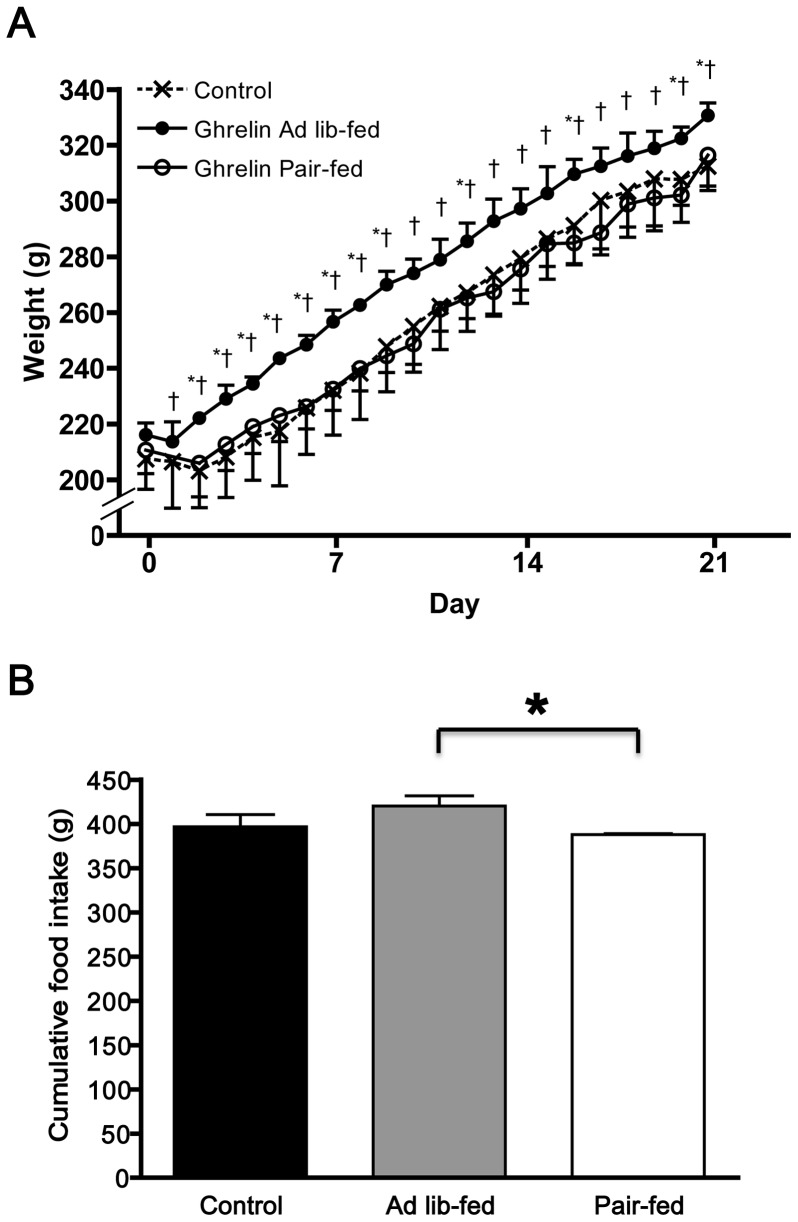
Effect of chronic ICV ghrelin infusion on (A) body weight and (B) food intake. Three groups of rats (4–6 per group) were infused for 21 days with saline or ghrelin (1.5 μg/day). Rats infused with ghrelin were *ad lib*-fed or pair-fed (to saline-infused rats). * P<0.05 vs. control; † P<0.05 vs. paired-fed. Data are presented as the mean±SEM.

Chronic ICV ghrelin infusion (1.5 μg/day for 21 days) significantly increased body weight in ghrelin *ad lib*-fed compared with ICV saline-infused control rats (331±4 g vs. 312±9 g, P<0.05). However, there was no difference in body weight between the control rats and ICV ghrelin-infused rats that were pair-fed the food intake of the control rats (ghrelin pair-fed) (316±11 g) (ANOVA F value = 4.21) (Fig. 1A). Although there was no significant difference in cumulative food intake between the ghrelin pair-fed and control groups, ghrelin *ad lib*-fed rats had significantly higher cumulative food intake compared with ghrelin pair-fed rats (P<0.05) (ANOVA F value = 2.86) (Fig. 1B).

### Chronic ICV ghrelin infusion increases bone mass

**Figure 2:**
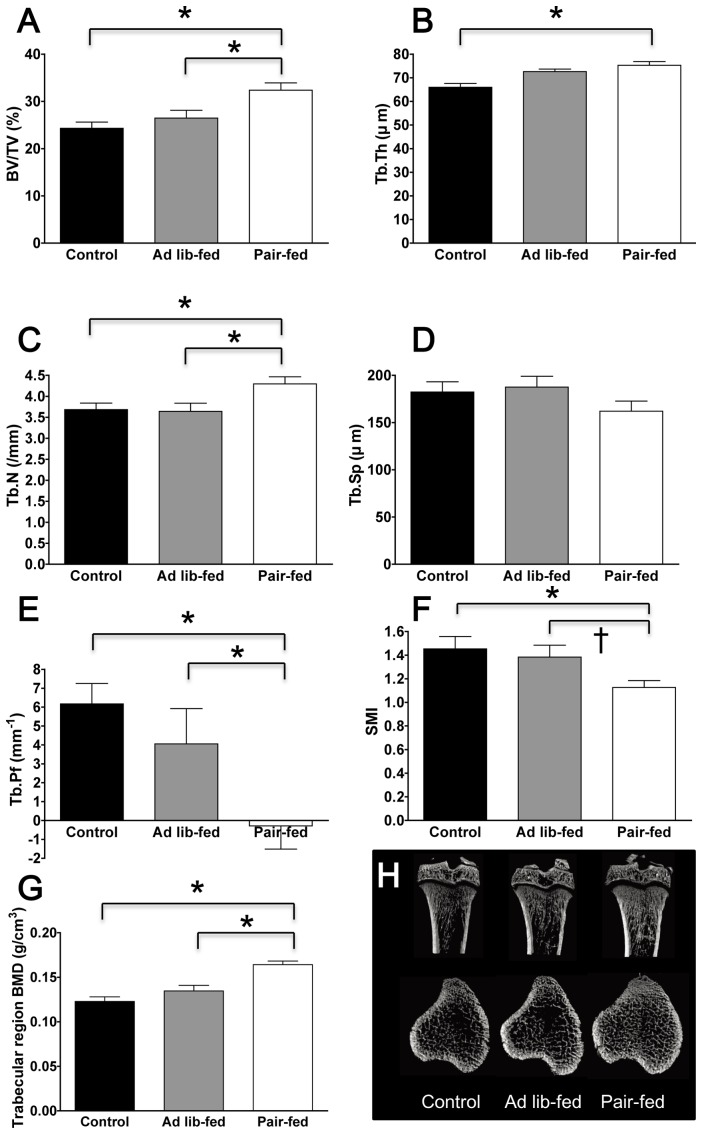
Effect of chronic ICV ghrelin infusion on the tibia trabecular bone phenotype. (A) Trabecular bone volume expressed as percentage of total tissue volume (BV/TV). (B) Trabecular thickness (Tb.Th). (C) Trabecular number (Tb.N). (D) Trabecular separation (Tb.Sp). (E) Trabecular pattern factor (Tb.Pf). (F) Structure model index (SMI); a lower SMI indicate plate-like structure, whereas a higher SMI indicate sphere-like structure. (G) Trabecular volumetric BMD. (H) Representative microCT images of the proximal tibia. Three groups of rats (4–6 per group) were infused for 21 days with saline or ghrelin (1.5 μg/day). Rats infused with ghrelin were *ad lib*-fed or pair-fed (to saline-infused rats). * P<0.05; † P<0.1. Data are presented as the mean ± SEM.

Chronic ICV ghrelin infusion significantly increased bone mass in ghrelin pair-fed group compared with control rats. This was indicated by increased bone volume percentage (BV/TV, 32.4±1.5% vs. 24.3±1.3%, P = 0.002) (ANOVA F value = 8.43), trabecular thickness (Tb.Th, 75.3±1.6 μm vs. 66.0±1.6 μm, P = 0.001) (ANOVA F value = 10.40), trabecular number (Tb.N, 4.3±0.2 mm^−1^ vs. 3.7±0.2 mm^−1^, P = 0.023) (ANOVA F value = 4.80), and volumetric BMD (0.164±0.004 g/cm3 vs. 0.123±0.005 g/cm3, P = 0.00005) (ANOVA F value = 20.18) of the tibia trabecular bone, as measured by microCT (Fig. 2). The trabecular pattern factor (Tb.Pf), a parameter inversely correlated to the connectivity, was significantly decreased in the ghrelin pair-fed group, indicating that the trabecular structure was more connected in the ghrelin pair-fed group (P<0.05, Fig 2) (ANOVA F value = 6.46). This result is consistent with the decreased structure model index (SMI) observed in the ghrelin pair-fed group, which suggests that the trabecular structure was more plate-like in the ghrelin pair-fed group, as compared with the rod-like structures in the control group (P<0.05, Fig. 2) (ANOVA F value = 4.45). There were no significant differences in bone mass observed between the control group and ghrelin ad-lib fed group. Similar trends were observed in femur and lumbar spine, with lower statistical significance (Figure S1 and S2). Cortical area (Ct.Ar) was significantly increased in the ghrelin *ad lib*-fed group compared to the control group (P<0.05, Figure S3) (ANOVA F value = 3.57). There was a tendency of increased cortical area fraction and cortical thickness in the ghrelin *ad lib*-fed group compared to the control group (P<0.1, Figure S3) (ANOVA F value = 1.73 and 2.49, respectively). There were no significant differences in *ex vivo* BMD measurements between the three groups (Table 1).

**Table 1:** Effect of chronic ICV ghrelin infusion on ex vivo BMD measurement of tibia, femur and spine by DXA.

	Control	Ghrelin Ad lib-fed	Ghrelin Pair-fed
Tibia ex vivo BMD (g/cm^2^)	0.119±0.002	0.125±0.004	0.127±0.003
Femur ex vivo BMD (g/cm^2^)	0.148±0.004	0.161±0.001 †	0.155±0.005
Spine ex vivo BMD (g/cm^2^)	0.154±0.002	0.158±0.003	0.157±0.003

* P<0.05. †P<0.1. Mean±SEM.

### Chronic ICV ghrelin infusion increases the mineral apposition rate

**Figure 3:**
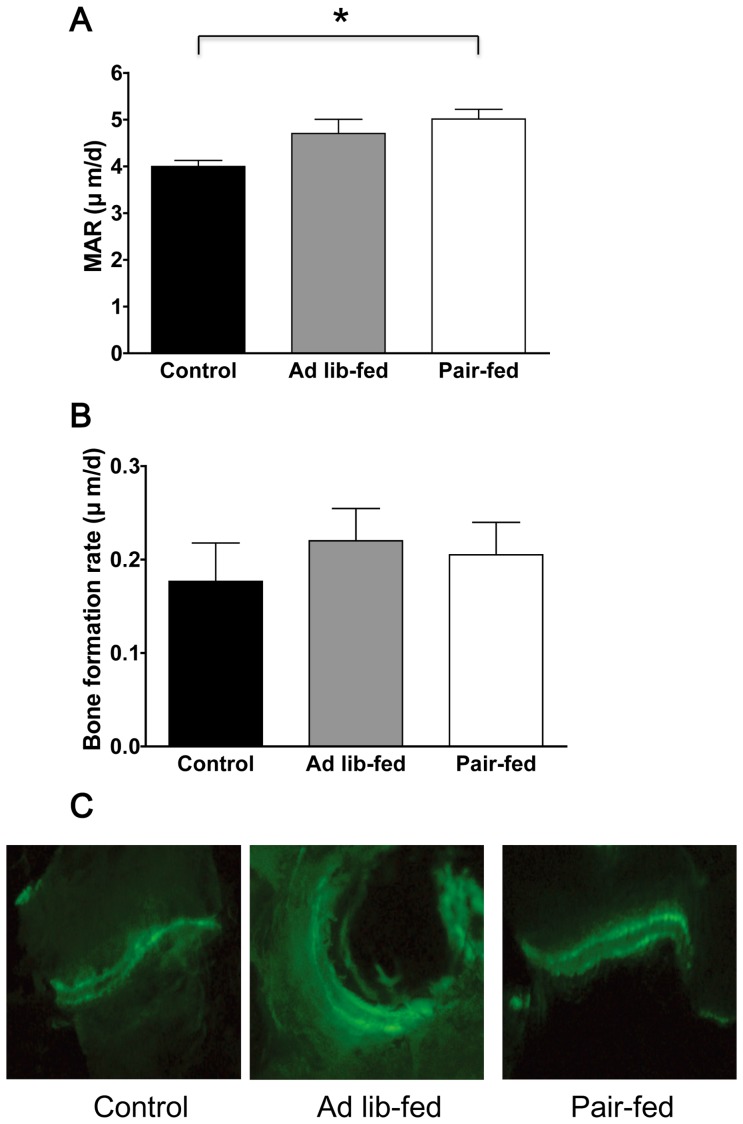
Effect of chronic ICV ghrelin infusion on the dynamic parameters of histomorphometric analyses. (A–B) Dynamic histomorphometric analyses of lumbar spine, including mineral apposition rate (MAR) and bone formation rate (BFR). (C) Representative fluorescent images obtained from lumbar spine after calcein double labeling (100× magnification). Three groups of rats (4–6 per group) were infused for 21 days with saline or ghrelin (1.5 μg/day). Rats infused with ghrelin were *ad lib*-fed or pair-fed (to saline-infused rats). * P<0.05; Data are presented as the mean ± SEM.

Chronic ICV ghrelin infusion significantly increased the mineral apposition rate (MAR) in the ghrelin pair-fed group compared with the control group (5.0±0.2 μm/d vs. 4.0±0.1 μm/d, P = 0.014, Fig. 3) (ANOVA F value = 5.36). The ghrelin *ad lib*-fed group tended to have a higher MAR compared with the control group (P = 0.073, Fig 3). No significant differences in bone formation rate were observed among the three groups.

### Effect of chronic ICV ghrelin infusion on serum markers

**Figure 4:**
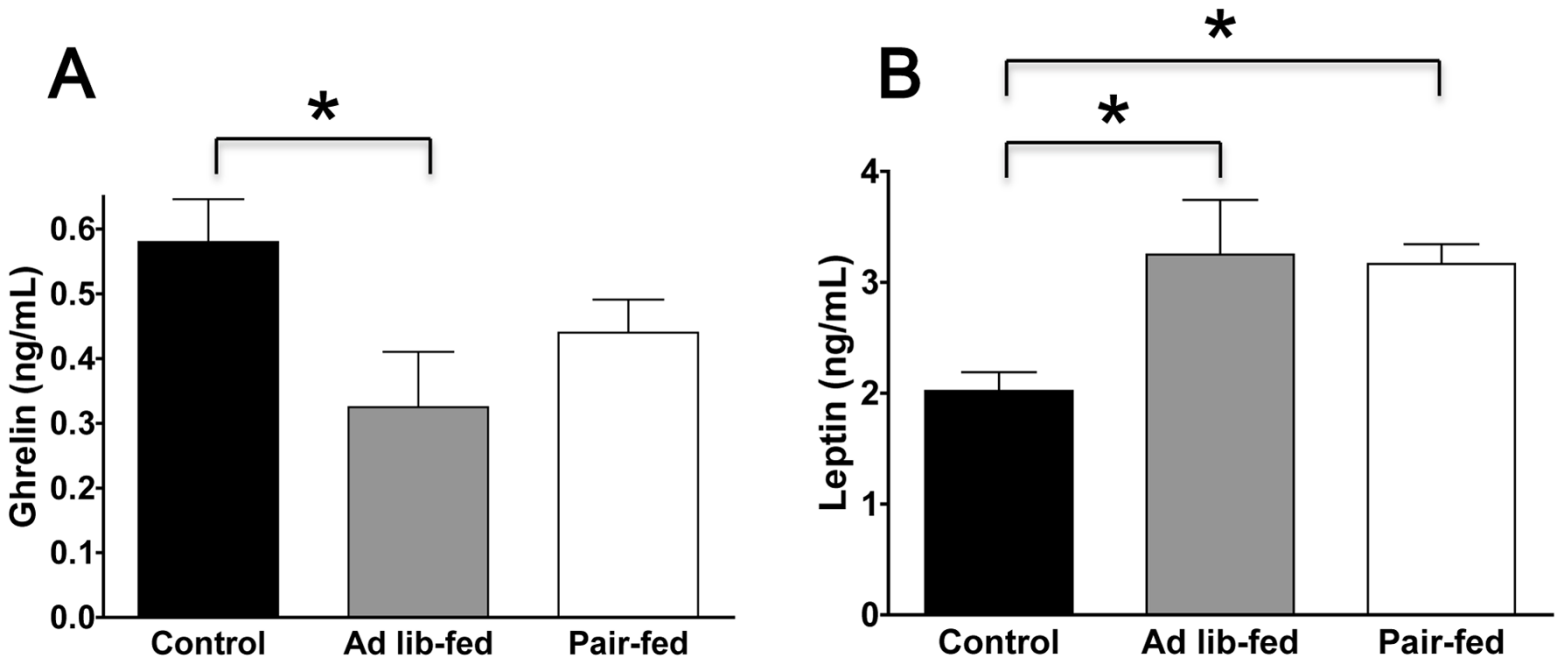
Effect of chronic ICV ghrelin infusion on serum ghrelin (A) and serum leptin (B). Three groups of rats (4–6 per group) were infused for 21 days with saline or ghrelin (1.5 μg/day). Rats infused with ghrelin were *ad lib*-fed or pair-fed (to saline-infused rats). * P<0.05. Data are presented as the mean ± SEM.

Chronic ICV ghrelin infusion significantly increased serum leptin in both the ghrelin *ad lib*-fed group and ghrelin pair-fed group compared with the control group (Fig. 4). Chronic ICV ghrelin infusion significantly decreased serum ghrelin in the ghrelin *ad lib*-fed group compared with the control group (P<0.05) (ANOVA F value = 3.55 and 6.31, respectively). However, there were no significant differences in IGF-1, CTX, TRAP-5b, and P1NP among the three groups.

## Discussion

Using a rat ICV administration model, we demonstrate that intracerebral infusion of ghrelin results in increased bone mass, connectivity, and mineral apposition rates in ghrelin pair-fed group. Chronic ICV administration of ghrelin increased weight gain in the ghrelin *ad lib*-fed group; however, this effect was abolished by pair feeding. These findings indicate that chronic central administration of ghrelin increased bone mass through mechanisms independent of changes in body weight. The duration of ICV ghrelin treatment (21 days) in this study could be considered relatively short to induce profound changes in bone mass. However, it should be noted that establishment of an animal model of chronic ICV treatment is technically challenging and the present study is the longest ICV ghrelin treatment attempted (21 days), with previous studies infusing ghrelin for only 6–12 days [8]–[10], [12]. Future study using Alzet osmotic pump model 2ML4 could extend chronic infusion up to 28 days.

Although we clearly demonstrated anabolic effects of ghrelin on bone (independent from ghrelin-induced effects on feeding), the mechanism for this effect is not known. One possible mechanism may be suppression of the sympathetic nervous system. Sympathetic activity has been reported to be suppressed by ICV administration of ghrelin [14] and stimulated by ICV administration of leptin [13]. Hypothalamic administration of leptin was reported to decrease bone formation due to the ability of leptin to increase sympathetic tone [5], [6], [17]. Numerous animal [5], [18] and human [19], [20] studies demonstrate that beta-blockers have protective effects on bone metabolism. Therefore, ICV ghrelin may increase bone mass through sympathetic suppression. Future studies using pharmacologic blockade of the sympathetic nervous system or local sympathectomy surgery will clarify this mechanism.

Other possible mechanisms include the secretagogue effect of ICV ghrelin on growth hormone [21]. However, the control group and ghrelin pair-fed group did not differ in serum IGF-1 levels so this does not support a role for growth hormone. These data are supported by a previous study that also reported no change in plasma IGF-1 after 7 days of ICV ghrelin treatment [9]. It has also been reported that ICV administration of ghrelin increased the plasma concentration of growth hormone on day 6 but this was not sustained on day 12 [22]. Therefore, it is unlikely that the effect of ICV ghrelin is mediated through a growth hormone-IGF-1 axis.

In contrast to the ghrelin pair-fed group, the ghrelin *ad lib*-fed group had a slight increase in cortical bone area and no significant difference in trabecular phenotypes compared with controls. However, the ghrelin *ad lib*-fed group did exhibit increased body weight gain. Therefore, the increased cortical bone mass could be the result of increased body weight and a consequent increase in mechanical loading on bone. Recently, we and others have reported that increased fat mass could have adverse effects on bone mass because of deleterious metabolic effects [23]–[27]. In addition, many researchers have suggested that weight gain and obesity could increase cortical bone mass through mechanical loading with a resultant decrease in trabecular bone due to metabolism or systemic effects [27]–[29]. Fatty acid lipotoxicity, numerous adipokines, inflammatory cytokines, aromatase, and insulin resistance have been suggested to mediate the deleterious effects of fat on bone metabolism [27]–[30]. Therefore, the absence of an increase in trabecular bone mass observed in the ghrelin *ad lib*-fed group could in part result from the deleterious effects of increased fat mass. The ghrelin *ad lib*-fed group had a significantly decreased serum ghrelin concentration, likely due to a compensatory response to increased body weight. And this decrease in serum ghrelin could result in decreased bone formation, which would then neutralize the anabolic effect of ICV ghrelin on bone.

Central ghrelin is reported to have an effect on adipose tissue independent from its effects on food intake [10], [11], [48]. Therefore, the ghrelin pair-fed rats could have some changes in adipose tissue deposition, which could be independent of food intake and weight change. These changes in adipose tissue deposition could have some direct and indirect effect on the bone phenotype of these ghrelin pair-fed rats. Furthermore, the ghrelin effect on adipose tissue deposition may have contributed to the lack of weight gain in the ghrelin pair-fed rats. However, we did not investigate the adipose tissue phenotypes in the present study to determine the role of adipose tissue deposition in the central regulation of bone metabolism.

In contrast to the increased bone mass determined by microCT measurement, there were no significant changes in *ex vivo* BMD measured by DXA in tibia, femur, and lumbar spine among the three groups. Several possible explanations may account for the discrepancy. DXA measurement reflects both trabecular and cortical bone mass, whereas microCT gives separate estimates of BMD for trabecular and cortical bone as well as reports volumetric mineral density in g/cm^3^. Therefore, DXA measurements cannot identify the difference in trabecular bone observed by microCT. It has been reported that the measurement precision of the excised femur BMD is generally not as precise as that for the intact femur BMD *in vivo,* although the general efficacy of *ex vivo* DXA measurements were acceptable [31].

Although similar trends were observed, the effects of ICV ghrelin on femur and spine were not statistically significant. There could be a skeletal site-specific effect of ICV ghrelin. Interestingly, a previous study reported a skeletal site-specific effect of leptin with lower femur BMD and higher spine BMD observed in leptin deficient mice [32]. However, no skeletal site-specific effect was observed in leptin receptor-deficient mice [7].

We measured serum ghrelin to investigate possible leakage of ghrelin into systemic circulation from the ICV injection. However, we found a significant decrease in serum ghrelin in the ghrelin *ad lib*-fed group as compared to the control group. This result indicated that leakage to systemic circulation was unlikely. This decrease in serum ghrelin and increase in serum leptin could be a compensatory response to the weight gain of the ghrelin *ad lib*-fed group. However, the increase in serum leptin in ghrelin pair-fed group is not due to the weight gain, since there was no weight gain in the ghrelin pair-fed group. Another possible explanation is that the increase in serum leptin observed in the ghrelin *ad lib*-fed group and ghrelin pair-fed group is likely a compensatory response related to ICV ghrelin injection. Previous studies have reported a similar trend of increased blood leptin levels in ICV ghrelin injected animals [9], [10]. Circulating ghrelin and leptin may contribute to bone metabolism via a direct effect on bone cells. Generally, ghrelin increases both osteoblast and osteoclast function [33]–[35] and leptin increases osteoblast function but decreases osteoclast function [36], [37]. However, these direct effects of ghrelin and leptin were inconsistent depending on the concentrations, assays, and cell types used [34], [38], [39]. Therefore, the role of serum ghrelin on bone metabolism is complex, and future study specifically aimed to investigate the role of serum ghrelin on bone metabolism is required to determine this issue.

We observed no significant differences in CTX, TRAP-5b, or P1NP, which are serum biochemical markers of bone turnover. Since ghrelin was placed in a subcutaneously transplanted osmotic pump, it is possible that after 21 days of incubation at body temperature, the effect of ghrelin was diminished due to peptide degradation. Therefore, the serum biochemical markers measured after 21 days of treatment may not have captured the dynamic bone metabolism state during the treatment period. However, other possibilities could be limitations from the ELISA assay or serum preparation procedure.

The findings of the current study have important clinical implications since ghrelin or ghrelin mimetics could be utilized as potential therapeutic modalities for osteoporosis. A cross-sectional study showed that serum ghrelin positively correlated with BMD [40]. In addition, there are a number of clinical trials investigating the effects of ghrelin or ghrelin mimetics on various conditions, including sarcopenia, cancer-related cachexia, anorexia nervosa, cystic fibrosis, postoperative gastric ileus, and gastroparesis [41]–[45]. These studies have already validated the safety and efficacy of ghrelin or ghrelin mimetics.

First limitation in the present study is the dose of ICV ghrelin treatment (1.5 µg/day). This dose is slightly higher than some studies (1.0 µg/day and 1.2 µg/day) [9], [46]. However, other studies have used higher doses (5–20 µg/day) [11], [47], [48]. The dose of present study (1.5 µg/day) could be insufficient or subthreshold to induce adequate central ghrelin effect. Second limitation was the statistical power of the present study. Most of the findings are based on LSD post hoc comparisons, which is very liberal. Additional post hoc comparisons with Scheffe and Tukey HSD test were performed and some of the findings (trabecular number, cortical bone area, serum ghrelin, body weight and food intake) did not reach statistical significance. And, it should also be noted that some of the findings with tendency of P<0.1 may be false positives. Third limitation was the conflicting fact that long duration of treatment was required to induce a sufficient bone metabolism change, whereas long duration of incubation in body temperature result in risk of ghrelin peptide degradation.

In conclusion, chronic central administration of ghrelin increases bone mass through a mechanism that is independent of body weight, suggesting that ghrelin may have a bone anabolic effect through the central nervous system.

## Supporting Information

Figure S1 **Effect of chronic ICV ghrelin infusion on the femur trabecular bone phenotype.** (A) Trabecular bone volume expressed as percentage of total tissue volume (BV/TV). (B) Trabecular thickness (Tb.Th). (C) Trabecular number (Tb.N). (D) Trabecular separation (Tb.Sp). (E) Trabecular pattern factor (Tb.Pf). (F) Structure model index (SMI). (G) Trabecular volumetric BMD. (H) Representative microCT images of the distal femur. Three groups of rats (4–6 per group) were infused for 21 days with saline or ghrelin (1.5 μg/day). Rats infused with ghrelin were *ad lib*-fed or pair-fed (to saline-infused rats). * P<0.05; † P<0.1. Data are presented as the mean ± SEM.
(TIF)

Figure S2 **Effect of chronic ICV ghrelin infusion on the spine trabecular bone phenotype.** (A) Trabecular bone volume expressed as percentage of total tissue volume (BV/TV). (B) Trabecular thickness (Tb.Th). (C) Trabecular number (Tb.N). (D) Trabecular separation (Tb.Sp). (E) Trabecular pattern factor (Tb.Pf). (F) Structure model index (SMI). (G) Trabecular volumetric BMD. (H) Representative microCT images of the lumbar spine. Three groups of rats (4–6 per group) were infused for 21 days with saline or ghrelin (1.5 μg/day). Rats infused with ghrelin were *ad lib*-fed or pair-fed (to saline-infused rats). * P<0.05; † P<0.1. Data are presented as the mean ± SEM.
(TIF)

Figure S3 **Effect of chronic ICV ghrelin infusion on the tibia cortical bone phenotype.** (A) Total cross-sectional area inside the periosteal envelope (Tt.Ar). (B) Cortical bone area (Ct.Ar). (C) Cortical area fraction (Ct.Ar/Tt.Ar). (D) Medullary area (Ma.Ar). (E) Cortical thickness (Ct.Th). (F) Cortical porosity (Ct.Po). (G) Periosteal perimeter (Ps.Pm). (H) Endocortical perimeter (Ec.Pm). (I) Representative microCT images of the mid-diaphysis tibia. Three groups of rats (4–6 per group) were infused for 21 days with saline or ghrelin (1.5 μg/day). Rats infused with ghrelin were *ad lib*-fed or pair-fed (to saline-infused rats). * P<0.05; † P<0.1. Data are presented as the mean ± SEM.
(TIF)
