# Supplementary figures and images for: Chronic Central Administration of Ghrelin Increases Bone Mass through a Mechanism Independent of Appetite Regulation
Source: PLoS One. 2013 Jul 2;8(7):e65505. doi: 10.1371/journal.pone.0065505 (PMC3699588; doi:10.1371/journal.pone.0065505)

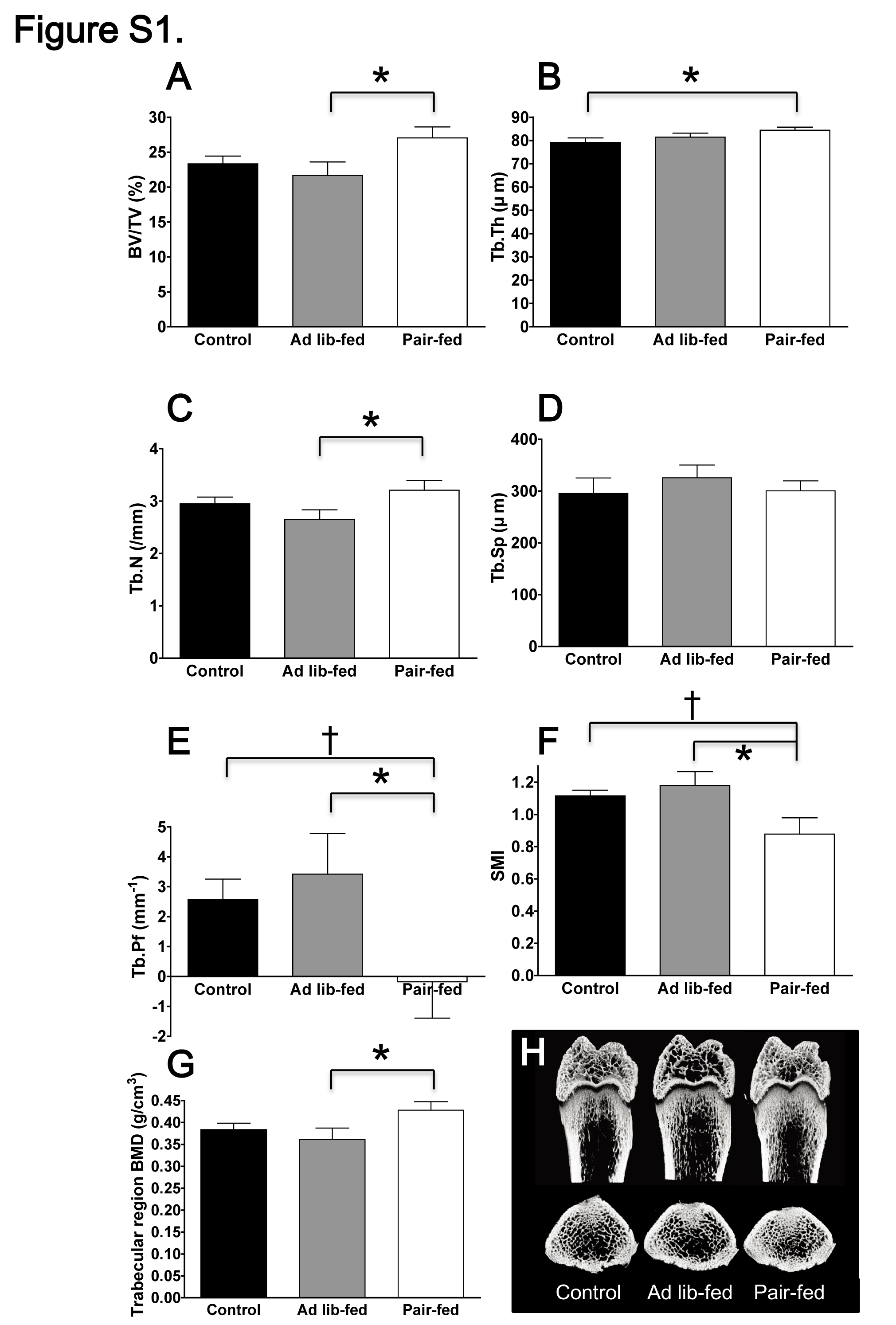

Supplement: Figure S1 — Effect of chronic ICV ghrelin infusion on the femur trabecular bone phenotype. (A) Trabecular bone volume expressed as percentage of total tissue volume (BV/TV). (B) Trabecular thickness (Tb.Th). (C) Trabecular number (Tb.N). (D) Trabecular separation (Tb.Sp). (E) Trabecular pattern factor (Tb.Pf). (F) Structure model index (SMI). (G) Trabecular volumetric BMD. (H) Representative microCT images of the distal femur. Three groups of rats (4–6 per group) were infused for 21 days with saline or ghrelin (1.5 μg/day). Rats infused with ghrelin were ad lib-fed or pair-fed (to saline-infused rats). * P<0.05; † P<0.1. Data are presented as the mean ± SEM. (TIF) [file pone.0065505.s001.tif]

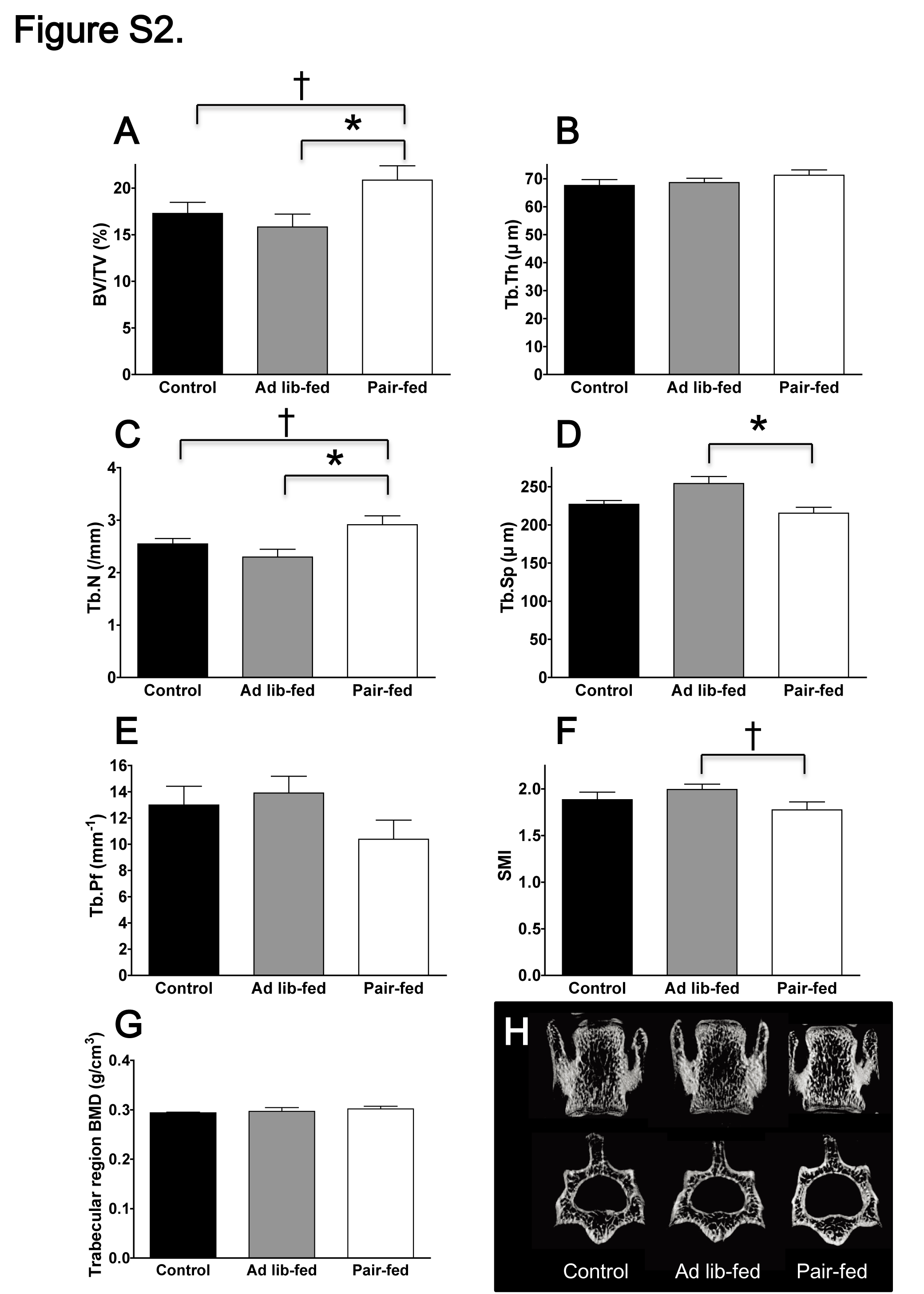

Supplement: Figure S2 — Effect of chronic ICV ghrelin infusion on the spine trabecular bone phenotype. (A) Trabecular bone volume expressed as percentage of total tissue volume (BV/TV). (B) Trabecular thickness (Tb.Th). (C) Trabecular number (Tb.N). (D) Trabecular separation (Tb.Sp). (E) Trabecular pattern factor (Tb.Pf). (F) Structure model index (SMI). (G) Trabecular volumetric BMD. (H) Representative microCT images of the lumbar spine. Three groups of rats (4–6 per group) were infused for 21 days with saline or ghrelin (1.5 μg/day). Rats infused with ghrelin were ad lib-fed or pair-fed (to saline-infused rats). * P<0.05; † P<0.1. Data are presented as the mean ± SEM. (TIF) [file pone.0065505.s002.tif]

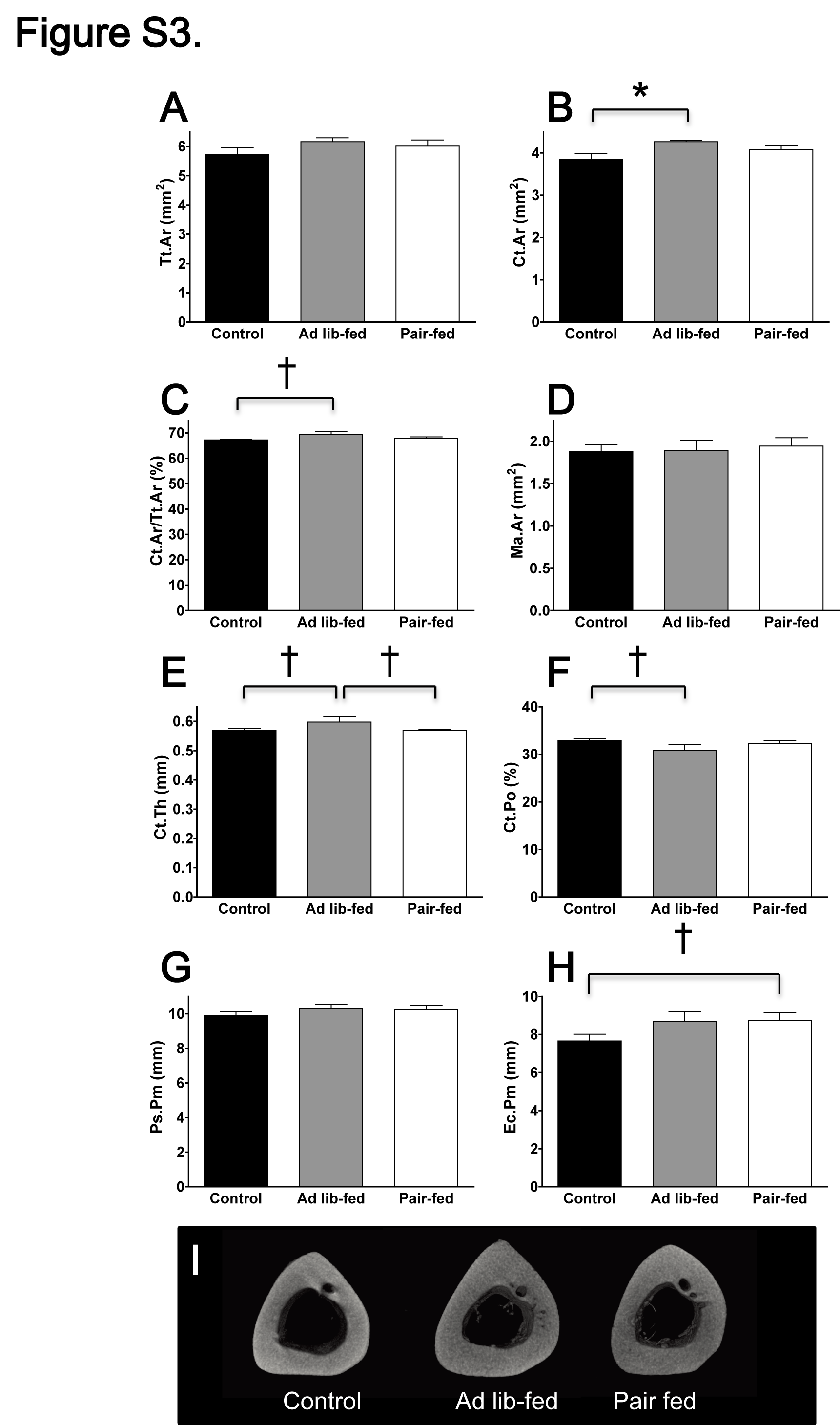

Supplement: Figure S3 — Effect of chronic ICV ghrelin infusion on the tibia cortical bone phenotype. (A) Total cross-sectional area inside the periosteal envelope (Tt.Ar). (B) Cortical bone area (Ct.Ar). (C) Cortical area fraction (Ct.Ar/Tt.Ar). (D) Medullary area (Ma.Ar). (E) Cortical thickness (Ct.Th). (F) Cortical porosity (Ct.Po). (G) Periosteal perimeter (Ps.Pm). (H) Endocortical perimeter (Ec.Pm). (I) Representative microCT images of the mid-diaphysis tibia. Three groups of rats (4–6 per group) were infused for 21 days with saline or ghrelin (1.5 μg/day). Rats infused with ghrelin were ad lib-fed or pair-fed (to saline-infused rats). * P<0.05; † P<0.1. Data are presented as the mean ± SEM. (TIF) [file pone.0065505.s003.tif]
